# Does repeat tibial tubercle osteotomy or intramedullary extension affect the union rate in revision total knee arthroplasty? A retrospective study of 74 patients

**DOI:** 10.3109/17453670903110683

**Published:** 2009-08-01

**Authors:** Byron E Chalidis, Michael D Ries

**Affiliations:** Department of Orthopaedic Surgery, University of CaliforniaSan Francisco, CAUSA

## Abstract

**Background and purpose** Tibial tubercle osteotomy (TTO) is an established surgical technique for exposing the stiff knee in revision total knee arthroplasty (RTKA). The osteotomy is usually performed through the anterior metaphyseal cancellous bone of the tibia but it can be extended into the intramedullary canal if tibial stem and cement removal are necessary. Furthermore, repeat osteotomy may be required in another RTKA. We assessed whether intramedullary extension of TTO or repeat osteotomy affected the healing rate in RTKA.

**Methods** We retrospectively evaluated 74 consecutive patients (39 women) with an average age of 60 (29–89) years who underwent 87 TTOs during RTKA. 1 patient had bilateral TTO. 10 patients had repeat TTO and 1 patient received 3 TTOs in the same knee. The osteotomy was extramedullary in 57 knees and intramedullary in 30 knees. Osteotomy repair was performed with bicortical screws and/or wires.

**Results** Bone healing occurred in all the cases. The median time to union was 15 (6–47) weeks. The median healing time for the extramedullary osteotomy group was 12 weeks and for the intramedullary osteotomy group it was 21 weeks (p = 0.002). Repeat osteotomy was not associated with delayed union. Neither intramedullary nor repeat osteotomy was found to increase the complication rate of the procedure.

**Interpretation** Reliable bone healing can be expected with intramedullary extension or repeat TTO in RTKA. However, intramedullary extension of the osteotomy prolongs the union time of the tibial tubercle.

## Introduction

The usefulness of tibial tubercle osteotomy (TTO) in revision total knee arthroplasty (RTKA) is well established (Whiteside 1995, Ries and Richman 1996, Bruce et al. 2000, Mendes et al. 2004, van den Broek et al. 2006, Young et al. 2008). A long osteotomy including the tibial tubercle and the proximal part of the anterior tibial crest provides a large bone surface for rigid fixation with multiple wires and/or screws. The osteotomized bone segment is maintained in continuity with the anterior compartment muscles, which preserves the vascularity at the osteotomy site and acts as a distal soft tissue tether resisting the quadriceps pull. Reliable bone healing has been reported with this technique in the majority of primary or revised knee arthroplasties (Whiteside 1995, Ries and Richman 1996, Bruce et al. 2000, Mendes et al. 2004, van den Broek et al. 2006, Young et al. 2008). However, occasional nonunion and proximal migration of the tibial tubercle or fracture of the tibial metaphysis can occur (Whiteside 1995, Mendes et al. 2004, Young et al. 2008).

The osteotomy is performed through the cancellous bone anterior to the intramedullary canal, leaving a broad area of bony contact between the osteotomized tibial tubercle and the host tibia. Occasionally, RTKA may require anterior exposure of the tibial canal for cement and stem removal. Intramedullary extension of the osteotomy permits direct access to the tibial prosthesis and surrounding cement but it is also associated with loss of cancellous bone at the osteotomy site. Thus, osteotomy union may be compromised as callus formation can only be achieved at the peripheral cortical bone.

For patients who have had a previous RTKA with TTO and require another RTKA, a second osteotomy at the same bone area may be necessary. Surgical dissection during the previous TTO may adversely affect the vascularity and healing potential of the second TTO, predisposing the patient to delayed union or nonunion. We investigated whether intramedullary extension or repeat TTO would affect the union and complication rate in RTKA.

## Methods

**Figure 1.**
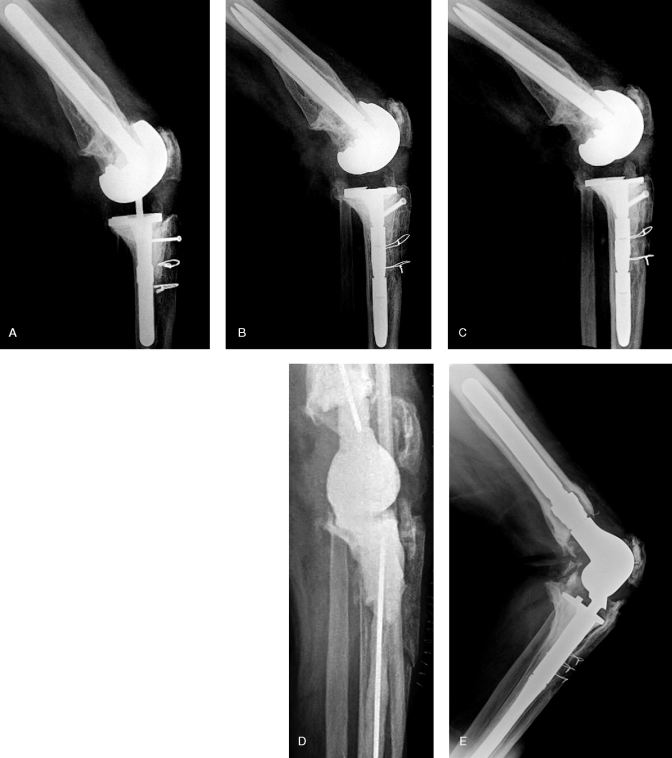
3 TTOs on the same knee. A. Healed primary TTO in a 57-year-old man who underwent RTKA of the left knee. A bicortical screw proximally and 2 wires distally were used for osteotomy fixation. B. 4 years later, implant loosening and instability developed, and another RTKA with a second TTO was performed. Fixation of the osteotomy was achieved with the same osteosynthesis method as the previous TTO. C. Complete consolidation of the osteotomized fragment was seen radiographically 6 months postoperatively. D. 2 years later, knee infection occurred. The implants were removed and an articulated antibiotic-impregnated cement spacer was inserted. A third TTO was performed during removal of the infected TKA and antibiotic cement spacer implantation. The osteotomy was left unfixed to avoid introduction of metallic fixation into a contaminated wound. E. Fixation of the osteotomy with 3 wires was performed during the second stage of RTKA. At the final follow-up 2 years postoperatively, the tibial tubercle was well healed.

We retrospectively evaluated 74 consecutive patients who underwent 87 TTOs during RTKA in a single-surgeon, single-institution setting. All the procedures were performed between November of 1997 and December of 2006. There were 35 men and 39 women with an average age of 60 (29–89) years. 62 patients underwent 1 TTO, 10 patients had repeat TTO, and 1 patient had 3 TTOs in the same knee (Figure 1). 1 patient also had bilateral TTO.

Osteoarthritis (54 knees) was the most common indication for the primary total knee arthroplasty (TKA), followed by posttraumatic arthritis (10 knees), rheumatoid arthritis (6 knees), hemophilic arthropathy (3 knees), septic arthritis (1 knee), and osteosarcoma of the distal femur (1 knee).

The reason for RTKA with TTO was staged treatment of infection in 38 knees, stiffness and arthrofibrosis in 21 knees, aseptic loosening in 15 knees, instability in 6 knees, patellar maltracking in 2 knees, polyethylene wear-through in 2 knees, femoral component malrotation in 1 knee, heterotopic ossification in 1 knee, and tibiofemoral dislocation in 1 knee.

Preoperative lag of extension and flexion contracture were present in 23 and 30 knees, respectively. In 8 knees, a rectus snip (6 knees) or a V-Y turndown procedure (2 knees) had been performed earlier during another RTKA. Overall, the average number of RTKAs per patient was 1.5 (1–7).

**Figure 2.**
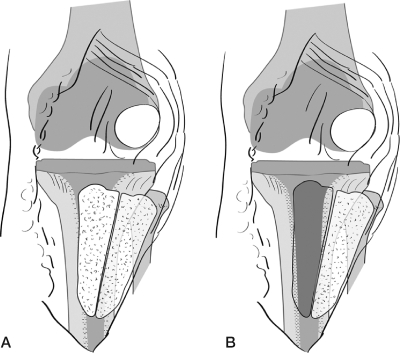
Intramedullary and extramedullary TTO A. Diagram illustrating a TTO elevated in a medial to lateral fashion. The anterior compartment muscles as well as the patellar tendon and patella are attached to the osteotomized bone fragment. The osteotomy is performed through the metaphyseal bone anterior to the tibial intramedullary canal, exposing cancellous bone on both sides of the osteotomy. B. Diagram illustrating extension of the osteotomy into the intramedullary canal to facilitate tibial stem and cement removal. Cancellous bone has been removed from the proximal tibia to expose the intramedullary canal.

Patients were scheduled to be evaluated clinically and radiographically preoperatively and postoperatively at 6 weeks, 3 months, 6 months, and annually thereafter—or at additional time intervals if residual symptoms and radiographic findings necessitated further examination. The average follow-up was 49 (6–108) months. None of the patients were lost to follow-up before healing of the osteotomy had occurred. Anteroposterior, lateral, and patellar radiographs were obtained from all the patients for the evaluation of implant position, patellar tracking, and status of the TTO. The measurement of length and width of osteotomy was done electronically with digitized lateral radiographs using imaging software. The osteotomy was classified as extramedullary when the bone cut was through the metaphyseal cancellous bone of the anterior tibia, or intramedullary when it extended more deeply from the inner surface of the tibial tubercle (Figure 2). The osteotomized bone fragment was considered to be healed to the host tibia when radiographic evidence of bridging callus formation was observed on the lateral radiograph.

### Surgical technique

The procedure was performed through a medial parapatellar arthrotomy and the medial proximal tibia was dissected subperiostally to facilitate tibial external rotation and lateral patellar subluxation. Retropatellar adhesions were released and scar tissue dissected from the medial and lateral gutters. If patellar subluxation was associated with excess tension in the extensor mechanism and there was risk of patellar tendon avulsion, a TTO was carried out. The tibial tubercle together with a segment of anterior tibial crest was elevated in a medial to lateral direction. The bone cut was initiated with a thin oscillating saw and completed with 2 broad osteotomes, leaving a long tibial bone fragment for late repair. In comparison with the previously described technique of Whiteside and Ohl (1990) in which the distal end of the bone cut was made in a transverse manner, the osteotomy was distally tapered to avoid a stress riser in the anterior tibial cortex. Similarly, no step-cut was done proximally and the osteotomy was extended to the knee articular surface. The osteotomized bone segment, along with the attached anterior compartment muscles and patellar tendon, was then hinged laterally to expose the knee.

At the completion of the RTKA, the tibial tubercle was reduced in its anatomic position or alternatively displaced up to 1 cm medially and/or proximally to achieve optimal quadriceps tension, knee flexion, and alignment of the extensor mechanism. Lateral release was performed routinely to improve or correct patellar alignment. Osteotomy repair was performed with bicortical screws (9 knees), Luque wires (16 knees), 1 screw and wires (52 knees) (Figure 1A-C), or 2 screws and wires (7 knees).

In infected knee arthroplasty, a 2-stage procedure was followed with a time interval of 6–8 weeks. The TTO was made either during reimplantation of the components (second stage, 33 knees) or at the time of implant removal and introduction of cement-spacer (first stage, 5 knees). In the latter group, the bone fragment with the muscular attachments remained unfixed (Figure 1D) until the second stage of RTKA (Figure 1E).

Postoperatively, no weight bearing or range of motion restrictions were applied and knee flexion exercises were started on the day after surgery.

### Statistics

Statistical evaluation was carried out with the the SPSS software package vesrion 16.0. Since data histograms showed skewed distribution of the variables, nonparametric methods of analysis were chosen. Data are presented as median and range. Any differences in union time between extramedullary and intramedullary or first and repeat osteotomy groups were examined using the Mann-Whitney rank-sum test. The Kruskal-Wallis statistic was calculated to test for a dependency between fixation technique and union time. The changes in knee range of motion, extensor lag, and flexion contracture before and after surgery were evaluated with the Wilcoxon signed-rank test. Statistical significance was assumed for a p-value of < 0.05 (or determined with use of a 95% confidence interval).

## Results

The median length and width of osteotomy was 106 (79–153) mm and 13 (8–25) mm, respectively. The tibial tubercle was re-attached medially in 16 cases and recessed proximally in another 29 cases.

Bone healing occurred in all cases. The median time to union was 15 (6–47) weeks. The osteotomy was extramedullary in 57 knees and intramedullary in 30 knees. The median healing time in the first group was 12 (6–47) weeks and in the second group it was 21 (7–38) weeks (p = 0.002, Mann-Whitney test).

The median union time for the first TTO was 15 (6–47) weeks and for the repeat TTO (including the knee with the 3-time osteotomy) it was 21 (7–27) weeks (p = 0.6, Mann-Whitney test). The fixation technique had no detectable effect on osteotomy union (p = 0.2, Kruskal-Wallis test).

The median range of motion (ROM) increased from 60 degrees preoperatively to 95 degrees postoperatively (p < 0.001, Wilcoxon test). Similarly, the median extensor lag improved from 10 to 5 degrees after surgery (p = 0.008, Wilcoxon test) and flexion contracture improved from 10 to 3 degrees (p < 0.001, Wilcoxon test) (Tables 1 and 2).

**Table 1.** Improvement in knee motion. Values are median (range).

Parameter	Preoperatively	Postoperatively	p-value (Wilcoxon test)
Flexion (degrees)	80 (0–135)	95 (40–135)	< 0.001
Extension (degrees)	0 (0–50)	0 (0–35)	< 0.001
Arc of motion (degrees)	60 (0–135)	95 (30–136)	< 0.001
Extensor lag (degrees)	10 (0–35)	5 (0–20)	0.008
Flexion contracture (degrees)	10 (0–50)	2.5 (0–35)	< 0.001

**Table 2.** Knee extensor lag and flexion contracture. Values are numbers of knees.

Range	Extensor lag		Flexion contracture	
	Preoperatively	Postoperatively	Preoperatively	Postoperatively
≤ 10 degrees	16	11	16	13
11–20 degrees	3	3	6	2
21–30 degrees	3	–	1	3
31–40 degrees	1	–	4	–
41–50 degrees	–	–	3	–
Total	23	14	30	18

Avulsion of the proximal part of the tibial tubercle occurred in 3 knees and superior migration of the entire osteotomized fragment was noted in 2 knees. The tibial tubercle fragment displacement was not evident on the immediate postoperative radiographs, but was identified at the time of routine follow-up, either at 6 weeks or 3 months postoperatively. Once displacement of the osteotomy had occurred, knee flexion beyond 100 degrees and quadriceps strengthening exercises were restricted for 3 months. The osteotomy was extramedullary in 3 of the knees mentioned above and intramedullary in the remaining 2. The amount of displacement ranged from 5 to 15 mm and the time to union in the 5 cases was 10, 16, 20, 21, and 47 weeks. 4 of the 5 patients were asymptomatic and had full active extension of the knee. In the last patient with a history of rheumatoid arthritis and steroid use, skin necrosis developed directly over the tibial tubercle, which was associated with 15 mm of superior migration of the proximal portion of the TTO. A medial gastrocnemius muscle flap transposition was preformed, and at the 1-year follow-up the patient had an arc of motion of 100 degrees with a 20-degree extensor lag, and walked with a cane.

Postoperative manipulation was required in 10 knees. Removal of screw(s) and/or wires due to skin prominence was undertaken in 5 patients after radiographic evidence of osteotomy healing. No other complications were reported. Proper patellar tracking was found in all the operated cases.

## Discussion

Tibial tubercle osteotomy during RTKA has provided good clinical results in most published cases. Van de Broek et al. (2006) reported that successful osteotomy healing was achieved in 37 of 39 RTKAs. An average increase of 12 degrees in ROM was also noted at a mean follow-up of 2.5 years. Young et al. (2008) observed bone union in all but 1 of 41 TTOs during RTKA. Knee flexion and extension were improved by an average of 18 degrees and 5 degrees, respectively. Bruce et al. (2000) found osteotomy healing in all RTKAs (10 cases) and an increase in mean ROM from 60 degrees preoperatively to 78 degrees postoperatively. The authors also reported that 3 knees with preoperative fixed flexion deformity of up to 40 degrees were substantially improved at a mean follow-up of 3 years. Similarly, Mendes et al. (2004) noted union of the tibial tubercle in all but 2 of 67 RTKAs. In addition, 4 of 5 patients who had had extensor lag preoperatively had no extension deficit at the latest follow-up evaluation. In our study, all osteotomies healed and the median knee flexion and range of motion showed an increase of 15 degrees and 35 degrees, respectively. 9 of 23 knees with extensor lag had no deficit after the TTO, while flexion contracture of more than 10 degrees was found only in 5 patients postoperatively compared to 14 patients preoperatively. These observations indicate that favorable clinical outcome can be expected after RTKA with TTO.

In our experience, osteotomy has also been effective in more complex or multiple revision knee arthroplasties. We routinely extended the TTO into the intramedullary canal when necessary, to allow direct exposure for removal of well-fixed long cemented tibial stems. We also performed repeat TTO during a later revision of the same knee if adequate exposure could not be achieved with a less extensile approach. Finally, we left the TTO unfixed for a period of 6–8 weeks until the second-stage treatment of infected TKA. Our results illustrate that these techniques can be associated with the same high union rates and low complication rates reported for TTO by other authors (Bruce et al. 2000, Mendes et al. 2004, Ries and Richman 1996, van den Broek et al. 2006, Whiteside 1995, Young et al. 2008).

Occasional tibial fracture or migration of the tibial tubercle can, however, occur after TTO. Whiteside (1995) pointed out that all the TTOs during 110 revision arthroplasties that were fixed with 2 or 3 wires had healed, although 3 tibial fractures and 2 cases with proximal migration of the tibial tubercle complicated the surgical procedure. Mendes et al. (2004) reported that the osteotomy had slipped proximally up to 2 cm in 13 of 67 knees, but no extensor lag was identified. The osteotomy was done in a proximal step-cut and distal bevel-cut fashion, and primarily fixed with wires. Furthermore, 2 patients sustained a tibial stress fracture as a result of mechanical weakening of the anterior tibia cortex. Van den Broek et al. (2006) observed also that 4 of 39 proximally step-cut osteotomies migrated superiorly. However, re-fixation of the tibial tubercle was carried out in only 2 of them. In our study, the tibial tubercle migrated proximally in 5 of 87 knees. Apart from 1 case with concomitant wound healing problems requiring muscle flap transposition, no extension lag occurred. These findings suggest that slight superior displacement of the tibial tubercle can occasionally occur after TTO, although this is not necessarily associated with extensor mechanism dysfunction.

Favorable results have been reported after fixation of the tibial tubercle by using either wires (Whiteside 1995, Barrack et al. 1998, Mendes et al. 2004, Young et al. 2008), screws (Wolff et al. 1989, Ries and Richman 1996, van den Broek et al. 2006), or both (Bruce et al. 2000). Van den Broek et al. (2006) reported that lag screws led to consolidation of the tibial tubercle within 6 weeks in 14 RTKAs, within 3 months in 16 knees, and within 6 months in 6 knees. Young et al. (2008) noted that the mean union time with use of 3 double-stranded Luque wires was 14 (8–24) weeks. Bruce et al. (2000) found that the average time to union at the proximal and distal ends of the osteotomy was 8 and 24 weeks, respectively, after fixation with wires. We found no difference in union time between the fixation groups, and healing occurred even after 1 or 2 previous osteotomies. Repeat osteotomy was successful without increasing the time to union or the incidence of tibial tubercle migration. We believe that preservation of the musculature sleeve is of primary importance for bone segment viability, and stability and a good result can be achieved after a previously performed TTO osteotomy by using either wire or screw fixation. This is further supported by the relative stability of the unfixed tibial tubercle in the staged treatment of infected TKA and the early callus formation at the osteotomy site, which was often observed during the second-stage reimplantation procedure. The TTO remained stable after insertion of a cement spacer, but in the absence of internal fixation probably because of the distal soft tissue attachment to the anterior compartment muscles.

The tibial tubercle segment is required to be of adequate size to facilitate secure hardware fixation and provide a wide contact area between the opposing bone surfaces. Wolf et al. (1989) found a high failure rate in osteotomies of less than 3 cm in length, which could not safely accommodate lag screws. However, more recent studies have not shown any major problems with longer osteotomies of more than 6–7 cm. Similarly, the depth of osteotomy has been considered an important factor for the success of the technique and values of approximately 1–2 cm, at the point of tibial tubercle, have been recommended for prevention of bone fragmentation and comminution (Ries and Richman 1996, van den Broek et al. 2006, Whiteside 1995).

We found that intramedullary extension of the bone cut is associated with an increase in the union time. We believe that this delay is related to the relatively small cortical bone-to-bone contact area and lack of cancellous bone apposition at the site of tibial osteotomy. However, bone union occurred in all cases requiring intramedullary extension of the osteotomy without any postoperative restrictions in mobility. The good healing capacity of TTO indicates that it can be safely extended into the intramedullary canal to allow access for cement and tibial stem removal.

The inherent limitations of this study include its retrospective design and the small number of patients. In addition, the evaluation of healing time was based on the relatively subjective criterion of radiographically bridging callus formation at the osteotomy site. Thus, the overall sensitivity and specificity of the method could not be clearly defined. Weight bearing activity was not quantitated. However, unrestricted knee motion and weight bearing after surgery did not appear to compromise the rate of osteotomy healing.

In conclusion, tibial tubercle osteotomy can be successfully performed more than once in RTKA. Intramedullary extension of the osteotomy provides adequate access to the tibial bone canal, but prolongs the union time.
